# Botulinum Toxin: Non-cosmetic Indications and Possible Mechanisms of Action

**DOI:** 10.4103/0974-2077.41148

**Published:** 2008-01

**Authors:** Uwe Wollina

**Affiliations:** *Department of Dermatology and Allergology, Hospital Dresden-Friedrichstadt, Academic Teaching Hospital of the University of Dresden, Friedrichstrasse 41, 01067 Dresden, Germany*

**Keywords:** Acetylcholine, Botulinum toxin, SNARE complex

## Abstract

Botulinum toxin (BTX) has gained a great interest in cosmetic dermatology for its effects on hyperkinetic facial lines. Understanding the basic research and analysis of effects of this potent drug can lead to other possible indications of interest for dermatologists. The use of BTX in focal hyperhidrosis is well established, but BTX has also effects on pain perception, itch and inflammation as discussed in this review.

## BOTULINUM TOXIN

Botulinum toxin (BTX) is produced by *Clostridium botulinum* as a complex mixture of neurotoxic polypeptides and non-toxic protein components. Seven different serotypes of neurotoxins have been identified- A,B,C,D,E,F and G. Synthesized as a single-chain polypeptide of ~150 kD, BTX has relatively little potency until it is cleaved by trypsin or bacterial enzymes into two chains, a heavy chain of 100 kD responsible for binding to the target structure and a light chain of 50 kD known as the toxifying chain. These two chains are linked together by a disulfide bond. The light chain of these neurotoxins contain a Zn^2+^-binding motif with enzymatic activity. BTX serotypes act as zinc-dependent endopeptidases. The best characterized toxins are botulinum toxin type A (BTXA) and botulinum toxin type B (BTXB), both used therapeutically and commercially available.[1,2]

## BTX: THE MOLECULAR MODE OF ACTION [FIGURE 1]

**Figure 1:**
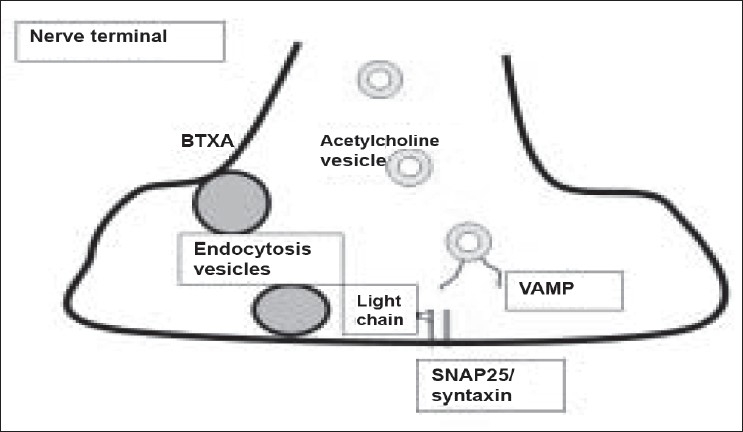
Synaptosomal acetylcholine vesicle fusion requires a complex interaction of VAMP and membrane bound SNAP25/syntaxin. BTXA is entering the nerve ending by endocytosis. The light chain of the molecule specifically cleaves SNAP-25. The target for some other BTX serotypes is different (see text). By this way, acetylcholine release from nerve endings is blocked.

When BTX is injected into a target tissue like a muscle or into the skin for anhidrotic effects, the heavy chain of the molecule binds to glycoproteins expressed specifically on cholinergic nerve endings. After internalization of the whole molecule by pinocytosis, the chains are cleaved within the cytoplasm. The light chain binds with high specificity to the soluble *N*- ethylmaleimide-sensitive fusion (SNARE) attachment protein receptors essential for exocytosis. SNARE is involved in transportation of acetylcholine from the cytosol into the synaptic cleft. It consists of a vesicle-associated protein (VAMP or v-SNARE, also known as synaptobrevin) and two target (t-SNARE) proteins: plasma membrane synaptosome associated protein (SNAP-25) and syntaxin. Recent studies have shown that the light chain exerts a multi-site substrate binding imparting the protease with exquisite specificity.[3]

Each of the various BTX molecules interacts with a specific part of SNARE. BTXA and botulinum toxin type E (BTXE) cleave SNAP-25 at different sites,[4] whereas BTXB, D and F cleave synaptobrevin.[5] Botulinum toxin type C (BTXC) cleaves both SNAP-25 and syntaxin.[6] The light-chain induced proteolytic cleavage of SNARE proteins inhibits the docking of acetylcholine vesicles on the inner surface of the nerve terminal and results in a blockade of vesicle fusion. When the target structure is a muscle, paresis with chemical denervation occurs. When the target tissue is a sweat gland the secretion is inhibited.

## BTX: DURATION OF MUSCULAR ACTION

Two to five days after BTX is injected into a muscle paresis occurs which lasts for at least 3 months until it gradually starts to wear off. The subjective duration of action in a given patient seems to be stable, but there is a great variability between different subjects.

Dose-effect correlations are known for electromyographic activity. Even very low doses produce some effect. By increasing the doses a plateau is reached where further dose increases will not result in stronger effects.

Dose-duration correlations exist to a lesser extent. Very low doses will produce effects more limited in time than higher doses. However, saturation will be seen with higher dosages at about 3 months.[7]

## BTX: EFFECTS ON STRIATED MUSCLES

Principally, muscular atrophy can be induced by usage of BTX for prolonged period of time, but it seems to occur very inconsistently. Muscular hypertrophy can be normalized with repeated injections of BTXA. There are some muscular targets which show extreme dose insensitivity with respect to the therapeutic outcome and adverse effects like blepharospasm or spasmodic dystonia.

BTX also affects the spinal stretch reflex of muscles. When a muscle is stretched, afferent signals from the muscle spindle organ are transmitted by Ia and II fibres. This does not only excite alpha motoneurons of the stretched muscle, but also the interneurons of the antagonistic muscle inhibiting alpha motoneurons to the antagonists. Gamma motoneurons of the stretched muscle are inhibited by alpha motoneuron collaterals. In animal models, it was demonstrated that BTX causes atrophy of both extrafusal and intrafusal muscle fibres of the biceps femoris of Wistar rats.[8] Gamma motoneurons of isolated rat masseter muscles could be blocked by BTX without affecting muscle strength.[9] The antidystonic effect of BTX may be explained by target muscle paresis and spinal reflex inhibition.

Investigations following a single BTX injection into the sternomastoid muscle of mice demonstrated the development of temporary nerve sprouts that eventually were capable of exocytosis with subsequent upregulation of adjacent nicotinic receptors of the muscle fibre, forming a functional synapse.[10] The kinetics of this process, however, cannot explain the longer duration of BTX efficacy for instance in hyperhidrosis.

## BTX: EFFECTS ON SMOOTH MUSCLES

BTX actions on hyperactive smooth muscles like sphincter Oddi or anal spincter - just to name only two of them - is mediated by action on the autonomic nervous system. BTX action on smooth muscle does not differ from the action on striated muscles.

## BTX: EFFECTS ON EXOCRINE GLANDS

BTX can be used to treat hyperactivity of sweat glands, lacrimal glands and salivary glands. This action is also mediated by inhibition of acetylcholine release.

The dose-effect relationship is different for each of these target tissues. The lowest dose needed to gain an anhidrotic effect is 2 U of Botox^®^ (BTA). The dose-response does not seem to be linear for sweat glands and the same seems to be true for the dose-duration effect. Very low doses (<10 U of Botox^®^) have a limited temporary effect with a relapse of 100% within 3 months in axillary hyperhidrosis. Intermediate doses of 50-100 U of Botox^®^ produce a duration of about 9-12 months, whereas higher doses of 200 U of Botox^®^ produce an anhidrotic effect in a part of patients than can last longer than 15 months.[11,12]

In patients suffering from Frey's syndrome, lower doses are needed as compared to axillary hyperhidrosis and longer durations have been obtained.[13]

## BTX: CENTRAL NERVOUS SYSTEM (CNS) EFFECTS

BTX cannot pass the blood-brain barrier because of its molecular weight of 150 kD. A retrograde axonal transport has been documented by radioactively labelled BTX in cats after intramuscular injection. Because the transport was so slow, it seems likely that BTXA was inactivated before reaching the CNS.[14] In cultured spinal cord cells, BTXA light chains persist for more than 11 weeks.[15]

On the other hand, BTX injection into muscles has indirect effects on CNS activity.[16,17]

## BTX: EFFECTS ON PAIN

Pain relief has been reported after injection of BTX into hyperactive muscles. In addition, in animal models muscular pain induced by formalin can also be reduced by BTXA suggesting a direct analgesic effect.[18] In rabbit iris muscles and dorsal root ganglia neurons, substance P, a neuropeptide involved in pain reception and neurogenic inflammation, can be blocked together with acetylcholine.[19–21]

Enkephalin gene expression is upregulated after BTX injection into the gastrocnemius muscle, while acidic fibroblast growth factor genes become downregulated.[22] Of all BTX serotypes, BTXA produced the strongest effects.

BTX also inhibits the neurotransmitter glutamate involved in nociception.[23] Furthermore, the release of calcitonin gene-related peptide (CGRP) in autonomic vascular nerve terminals[24] and noradrenaline in PC12 cells[25] by BTX have been demonstrated.

In myofascial pain syndrome, 10-20 U BTXA improved pain pressure thresholds, pain scores and visual analogue scores more effectively than dry needling.[26]

In an experimental setting in humans, intracutaneous application of 100 U BTXA (Dysport) was compared with pure saline. Ultraviolet-B induced sunburn spots were used as an inflammatory model. BTXA did not have an effect on inflammed and non-inflammed human skin as measured by heat pain threshold, cold pain threshold, area of secondary hyperalgesia and mechanical sensitivity.[27]

## BTX: ACTION ON PRURITUS

Itch is a sensation different from pain. In skin cutaneous, C-fibres conduct itch. They are sensitive to neurotransmitters, histamine and other inflammatory mediators like substance P, vasoactive intestinal peptide and CGRP. Prostaglandin E2, bradykinin, serotonin and interleukin-2 are known to produce itch when injected intradermally.[28]

Observations of an anti-pruritic activity of BTXA come from clinical investigations in lichen simplex[29] and dyshidrotic hand eczema.[30,31] In a prospective trial with eight patients with dyshidrotic hand eczema and left-right comparison (one hand with topical steroids, the other with steroids and 100 U of Botox^®^) showed that pruritus disappeared more quickly on the BTX hand.[30] The same observation was made in a study on 10 patients with dyshidrotic hand eczema. Itch as measured by visual analogue scale was reduced by BTXA by 39% *vs*. an increase of itch on the untreated side by 52%.[31] The exact mechanisms are yet not known, but inhibition of substance P and CGRP might be involved.

## BTX: IMMUNE EFFECTS

While most patients continue to respond to repeated BTX treatments, some become unresponsive as a result of the development of neutralizing or blocking antibodies. Only antibodies against the heavy chain block the function of the neurotoxin.[32] Immunization seems to be a higher risk in younger patients receiving higher doses more frequently intramuscular like in neuromuscular diseases.[33] There has been no report so far on the induction of neutralizing or blocking antibodies in patients where BTX has been used for treatment of hyperhidrosis or for aesthetic reasons.[34]

## SUMMARY

With better understanding of the mechanism of actions of BTX, new uses are being recognized and hence the indications for the use of BTX have expanded. However, it is important that the physician understands the anatomy and physiology and the pharmacology of BTX properly to derive maximum benefit for the patient.
